# The impact of pelvic radiotherapy on the gut microbiome and its role in radiation-induced diarrhoea: a systematic review

**DOI:** 10.1186/s13014-021-01899-y

**Published:** 2021-09-25

**Authors:** Lina Wang, Xiaohu Wang, Guangwen Zhang, Yan Ma, Qiuning Zhang, Zheng Li, Juntao Ran, Xiaoming Hou, Yichao Geng, Zheng Yang, Shuangwu Feng, Chengcheng Li, Xueshan Zhao

**Affiliations:** 1 Department of Radiation Oncology, The First Hospital of Lanzhou University, Lanzhou, China; 2 The First School of Clinical Medicine, Lanzhou University, Lanzhou, China; 3 Department of Radiation Medicine, Biomedical Centre, Institute of Modern Physics, Chinese Academy of Sciences, Yanbei Road, Chengguan District, Lanzhou City, 730000 Gansu Province China; 4 Affiliated Hospital of Gansu University of Chinese Medicine, Lanzhou, China; 5 Department of Radiation Therapy, Lanzhou Heavy Ion Hospital, Lanzhou, China

**Keywords:** Radiotherapy, Gastrointestinal microbiome, Diarrhoea, Systematic review

## Abstract

Pelvic radiotherapy is the key treatment for pelvic malignancies, usually including pelvic primary tumour lesions and lymphatic drainage areas in the pelvic region. Therefore, the intestinal tract in the radiation field is inevitably damaged, a phenomenon clinically referred to as radiation enteritis, and diarrhoea is the most common clinical symptom of radiation enteritis. Therefore, it is necessary to study the mechanism of radiation-induced diarrhoea. It has been found that the gut microbiome plays an important role in the development of diarrhoea in response to pelvic radiotherapy, and the species and distribution of intestinal microbiota are significantly altered in patients after pelvic radiotherapy. In this study, we searched for articles indexed in the Cochrane Library, Web of Science, EMBASE and PubMed databases in English and CNKI, Wanfang data and SINOMED in Chinese from their inception dates through 13 March 2020 to collect studies on the gut microbiome in pelvic radiotherapy patients. Eventually, we included eight studies: one study report on prostatic carcinoma, five studies on gynaecological carcinoma and two papers on pelvic carcinomas. All studies were designed as self-controlled studies, except for one that compared toxicity to nontoxicity. The results from all the studies showed that the diversity of intestinal flora decreased during and after pelvic radiotherapy, and the diversity of intestinal flora decreased significantly in patients with diarrhoea after radiotherapy. Five studies observed that the community composition of the gut microbiota changed at the phylum, order or genus level before, during, and after pelvic radiotherapy at different time points. In addition, the composition of the gut microbiota before radiotherapy was different between patients with postradiotherapy diarrhoea and those without diarrhoea in five studies. However, relevant studies have not reached consistent results regarding the changes in microbiota composition. Changes in the intestinal flora induced by pelvic radiotherapy and their relationship between changes in intestinal flora and the occurrence of radiation-induced diarrhoea (RID) are discussed in this study, providing a theoretical basis for the causes of RID after pelvic radiotherapy.

## Background

More than 50% of cancer patients receive radiotherapy for cancer treatment [1, 2]. Pelvic irradiation has long been used as a curative or palliative therapy and has been proven successful for the treatment of various types of pelvic cancers, including cervical cancers, prostate cancers, and colorectal cancers. External beam radiotherapy is the most common type of radiotherapy and usually involves the primary tumour and regional lymph nodes in the pelvic region, ranging from the inferior border of the fifth lumbar vertebra to the superior border of the pubic symphysis and para-aortic lymph nodes, if necessary[3]. Therefore, in patients undergoing pelvic radiotherapy, damage to normal organs and tissues in the pelvic region is inevitable during treatment. Among these normal organs, the intestinal bowel is one of the most radiation-sensitive organs, and radiation injury inevitably occurs in the intestine in the radiation field.

Clinically, radiation-induced intestinal damage is collectively referred to as radiation enteropathy (RE), and diarrhoea is the most common RE-related symptom and is known as radiation-induced diarrhoea (RID). Acute diarrhoea occurs more than 2–3 weeks after pelvic radiotherapy, manifesting as an increased frequency of loose watery stool, abdominal pain, and bloating. Late injuries occur months to years after radiotherapy and are characterized by intermittent diarrhoea, bloody stools, indigestion, severe intestinal stenosis, and ulceration. In terms of the incidence of RID, approximately 30–50% of patients have been reported to experience pelvic RID [4], with a higher incidence observed in patients treated with concurrent chemotherapy [5]. In the large EORTC 22,921 trial that investigated preoperative and postoperative therapies for rectal cancer, the incidence of grade 2 or higher diarrhoea was 17% in patients receiving preoperative radiation therapy (45 Gy in 25 fractions) and 34% in patients receiving concurrent infusion of 5-fluorouracil(5FU) [6]. Even with the current advanced intensity-modulated radiation therapy (IMRT), the occurrence of radiation-induced diarrhoea has not been effectively reduced. In the RTOG1203 study, acute toxicity and health-related quality of life were compared between patients with cervical and endometrial cancer reported during treatment to standard pelvic radiotherapy or intensity-modulated radiotherapy (IMRT). It was found that at the end of radiotherapy, 51.9% of women receiving standard radiotherapy and 33.7% of women receiving intensity-modulated radiotherapy experienced frequent or persistent diarrhoea, while 20.4% of patients receiving standard radiotherapy took antidiarrheal drugs 4 or more times a day [4]. Therefore, RID not only further impairs quality of life but also leads to interruption and delays in the radiotherapy process, resulting in suboptimal treatment.

The mechanisms underlying RID development after pelvic radiotherapy are becoming clearer. Previous evidence suggests that the intestinal microbiome plays an important role in the development of RID during and following cancer radiotherapy. Crawford and Gordon discovered that germ-free mice were resistant to lethal radiation injury and exhibited reduced radiation-induced epithelial cell damage than conventional mice with commensal gut microbial flora [7]. The overgrowth of Gram-negative bacilli was shown to be essential in the pathogenesis of RE [8]. Moreover, it was recently reported that bowel irradiation may lead to a general decrease in the gut microbiota, an imbalance in the gut bacterial community structure, and subsequent pathogenic effects on the epithelial mucosa [9]. Therefore, there is a clear link between gut microbiome composition and RID pathological states. An improved understanding of the pathogenesis of RID is required to develop and implement optimal preventive and curative approaches for patient treatment. This review summarizes existing clinical research on the influence of pelvic radiotherapy on the intestinal microbiome and the role of the gut microbiome in RID and discusses potential implications for clinical practice.

## Methods

### Literature identification

In this systematic review, the Preferred Reporting Items for Systematic Reviews and Meta-analysis (PRISMA) guidelines were used to ensure transparent and complete reporting, and the review protocol was registered on the International Prospective Register of Systematic Reviews (PROSPERO) database with registration number CRD42019128210.

### Data sources and searches

We searched for articles indexed in the Cochrane Library, Web of Science, EMBASE and PubMed databases in English and CNKI, Wanfang data and SINOMED in Chinese from their inception dates through 13 March 2020. Two researchers searched independently (Lina Wang and Qiuning Zhang), and one additional researcher resolved any possible controversies (Juntao Ran). Search terms and their combinations were employed as follows: (‘intestine flora' OR 'gut microflora' OR 'gut microbio*' OR 'intestinal microbio*' OR 'microbiota' OR 'gastrointestinal microbio*' OR 'intestinal micro flora' OR 'gastrointestinal flora' OR 'gut flora' OR 'gastrointestinal microbial communit*' OR 'intestinal micro ecology' OR 'enteric bacteria') AND ('radiation' OR 'radiotherapy' OR 'irradiation'). “Appendix” presents an example of the full electronic search strategy for the PubMed database. For a more comprehensive catalogue of studies, we also conducted a general probe of search engines and references of the included papers. Contacts were made with the authors of the papers when further information was needed.

### Study selection

Studies on the gut microbiome in pelvic radiotherapy patients were included. Cell and animal studies, case reports, research protocols, or studies including single subjects and expert comments were excluded. Studies using probiotics as an intervention were excluded due to the Cochrane Collaboration's published protocol for a systematic review assessing the effect of probiotics on the prevention or treatment of chemo- or radiotherapy-related diarrhoea in patients with cancer [10]. Inclusion criteria were defined using the following components: patient population (P): patients treated with pelvic irradiation, exposure of interest (I): pelvic radiotherapy, comparison (C): before, during, and after pelvic radiotherapy, outcome (O): the change in the gut microbiome following pelvic radiotherapy treatment and study designs of interest (S): randomized controlled trials (RCTs), prospective observational cohort studies, and retrospective studies.

### Data extraction

Two reviewers (Lina Wang and Qiuning Zhang) used a standardized form to independently extract and summarize the following data: first author, year of publication, study ID, region, cancer type, study design, total number of patients, number of diarrhoea patients, treatment dose, follow-up time, species of intestinal flora, changes in intestinal flora (before, during and after radiotherapy), evaluation criteria of diarrhoea, and grade of diarrhoea.

### Quality assessment

Two reviewers (Guangwen Zhang and Yan Ma) assessed the risk of bias based on the original study, possible updated studies, and supplementary materials, using a tool recommended by the Newcastle–Ottawa Scale (NOS) for cohort and case–control designs with an overall quality score ranging from 0 (minimum) to 9 (maximum) stars [11]. If a cohort study was scored as < 5, it was considered low quality. If, on the other hand, a cohort study scored ≥ 5, it was considered high quality. All disagreements in study selection, data extraction, and quality assessment were resolved by discussion to reach a consensus.

## Results

### Study selection

**Fig. 1:**
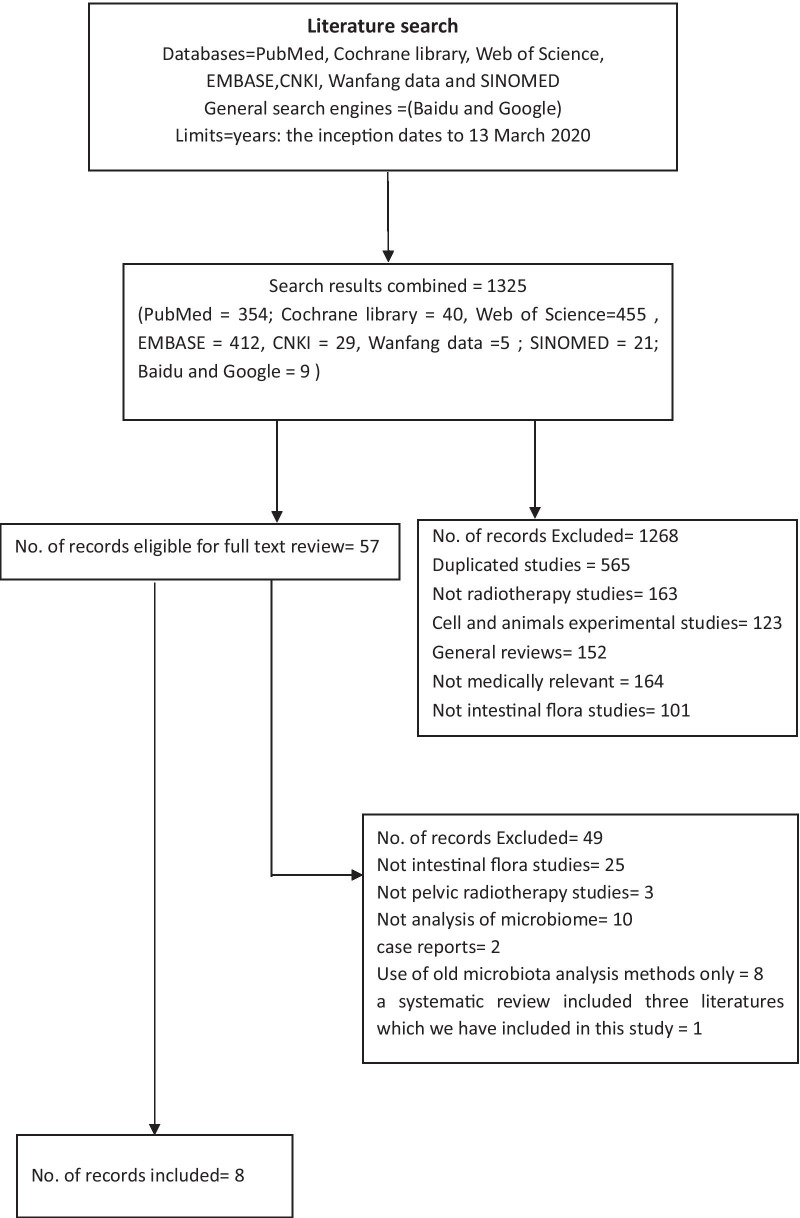
Study selection

Figure 1 shows a flowchart of the study selection procedure. A total of 1325 related studies were screened for eligibility, and 1268 studies were excluded after browsing the titles and abstracts. Forty-nine articles were subsequently excluded after reviewing the full text, three of which were articles on changes in the intestinal microbiota caused by nonpelvic radiotherapy. One of the reports on gynaecological cancer was a systematic review [12], and it included three studies that we included in this study. Therefore, it was excluded. Ultimately, eight studies were included [13–20]: one study on prostatic carcinoma, five studies on gynaecological carcinoma and two papers on pelvic carcinomas. One study was a conference paper on cervical cancer.

### Study quality

The median quality score of the studies was 7.375 (range 5–8). There were six articles with eight stars, one with six stars, and one with five stars. All included articles were considered high quality. The results are shown in Table 1.

**Table 1:** The results of literature quality evaluation

Study	Selection				Comparability	Outcome			Quality score
	1	2	3	4	1	1	2	3	
Mitra 2020		*	*	*	**	*	*	*	8
Wang 2019		*	*	*	**	*	*	*	8
Reis Ferreira 2019		*	*	*	**	*	*	*	8
Medrano 2017		*	*	*	*	*			5
Wang 2015		*	*	*	**	*	*	*	8
Nam 2013		*	*	*	**	*	*	*	8
Manichanh 2008		*	*	*	**	*	*	*	8
Cuzzolin 1992		*	*		**	*	*		6

The primary terms of NOS included selection of patients, comparability, and assessment of outcome. The selection section has four questions: (1) representativeness of the exposed cohort; (2) selection of the non-exposed cohort; (3) ascertainment of exposure; (4) demonstration that outcome of interest was not present at start of study. The comparability section has one question: (1) comparability of cohorts on the basis of the design or analysis. The outcome section has three questions: (1) Assessment of outcome; (2) was follow-up long enough for outcomes to occur; (3) adequacy of follow up of cohorts

### The main characteristics of the included studies

The main characteristics of these studies along with the details of the study design are listed in Table 1. All studies were on the relationship between pelvic radiotherapy and changes in intestinal microbiota, including one retrospective study and seven prospective cohort studies. The number of patients in the experimental group receiving pelvic radiotherapy and the number of patients in the healthy control group in the included studies are listed in Table 2. Six studies reported pelvic radiation doses, between approximately 40 and 74 Gy, and one study on prostatic carcinoma reported specific target volumes for pelvic radiotherapy. All studies used 16S rRNA technology, except for one that used bacterial culture and counting technology. Six studies collected stools from patients for testing, and two studies collected rectal or sigmoidal specimens for testing. The criteria for evaluating pelvic radiation-induced diarrhoea in the included studies were inconsistent, which prevented us from conducting a quantitative study on the correlation between diarrhoea and microbiota. All studies were designed as self-controlled studies, except for one that compared toxicity to nontoxicity. Although the time points for the detection of microbiota were not consistent, detection was basically performed before radiotherapy, during radiotherapy and after radiotherapy in these self-controlled studies.

**Table 2:** The main characteristics of the included studies

First Author	year	Type of study	Type of disease	No. of subjects	No. of healthy controls	RT dose (Gy)	Analysis technique microbiome	sample	Evaluation criteria of diarrhea	No of patients reporting diarrhoea	Before–after design	CCRT* or not	No. of testing time point	testing time point
Mitra	2020	Prospective cohort	Cervical cancer	35	0	–	16 s-rRNA	Stool	EPIC	–	Yes	Yes	4	Before RT
Wang	2019	Prospective cohort	Cervical cancer	18	0	50.4 Gy	16 s-rRNA	Stool	–	10	No	No	2	One day before RT,
Reis Ferreira	2019	Prospective cohort	Prostate cancer	32	6	60–74 Gy	16 s-rRNA	Stool sigmoid /rectum mucosa	RTOG	_–_	Yes	_–_	6	Before RT,
Medrano	2017	Prospective cohort	Cervical cancer	20	20	_–_	16 s-rRNA	Rectum	EPIC	_–_	Yes	_–_	4	Before RT,
Wang	2015	Retrospective study	Cervical cancer; anal canal cancer and rectal cancer	11	4	44-50 Gy	16 s-rRNA	Stool	CTCAE	5	Yes	No	2	Before RT,
Nam	2013	Prospective cohort	Cervical cancer and endometrial cancer	9	6	50.4 Gy	16 s-rRNA	Stool	Diarrhea Indices	–	Yes	–	4	Before RT,
Manichanh	2008	Prospective cohort	Cervical cancer, endometrial cancer and rectal cancer	10	5	43.2–54 Gy	16 s-rRNA	Stool	CTCAE	6	Yes	_–_	4	Before RT,
Cuzzolin	1992	Prospective cohort	Gynecologic cancer	15	15	40 Gy	Bacterial culture and counting	Stool	–	–	Yes	No	5	Before RT,

*EPIC* The Expanded Prostate Cancer Index Composite questionnaire, *CTCAE* common terminology criteria for adverse events, *RT* radiotherapy, *CCRT* concurrent chemoradiotherapy, *RT* radiation therapy

– Not mentioned in the text

### Outcomes of the included studies

All studies that used 16S RNA technology to detect microbiota reported the results of microbiota diversity analysis. The microbiota diversity results included an index to calculate the community richness of the microbiota (observed species, Chao index, ACE analysis) and an index to calculate the community diversity of the microbiota (Shannon index, Simpson, Coverage). There were differences in the detection indicators in each study. However, the results from all studies demonstrated that the diversity of the intestinal flora decreased during and after pelvic radiotherapy, and the diversity of intestinal flora decreased significantly in patients who experienced diarrhoea after radiotherapy (see Table 3).

**Table 3:** Outcomes of microbiota changes in the included studies

First Author	year	Detection method	Results of microbiota diversity analysis	Increased intestinal flora after RT		Decreased intestinal flora after RT		Increased intestinal flora before RT in diarrhea		Decreased intestinal flora before RT in diarrhea	
				Phylum	Genus	Phylum	Order/genus	Phylum	Genus/class	Phylum	Genus/class
Mitra	2020	16 s-rRNA	After RT, the diversity of microbiota decreased in patients with diarrhoea	–	Phascolarctobacterium Lachnospiraceae Veillonella Erysipelotrichaceae	–	Order: Clostridiales	–	Class: Sutterella, Finegoldia, Peptococcaceae (Clostridia)	–	Clostridiales
Wang	2019	16 s-rRNA	Patients with diarrhoea present reduced α‐diversity but increased β‐diversity of microbiota	–	–	–	–	Proteobacteria	Class: Gammaproteobacteria Genus: Serratia, Prevotella_9 Coprococcus Desulfovibrio	Bacteroidetes Firmicutes	Genus: Bacteroides, Blautia, Ruminococcaceae_UCG‐003
Reis Ferreira	2019	16 s-rRNA	After RT, the diversity of microbiota decreased in patients with diarrhoea	–	Roseburia Clostridium IV Phascolarctobacterium	–	–	–	Genus: sutterella	–	Genus: Roseburia
Medrano	2017	16 s-rRNA	After RT, the diversity of microbiota decreased and intestinal function decreased	–	–	–	–	–	–	–	–
Wang	2015	16 s-rRNA	The diversity of microbiota was lower in the diarrhoea group than in the no-diarrhoea and control groups	Unclassified bacteria	Bacteroides Clostridium_XIVa	Firmicutes Bacteroidetes	Genus: Faecalibacterium Lachnospiracea Oscillibacter Roseburia Streptococcus	–	Genus: Bacteroides, Veillonella, Dialister,	–	Genus: Clostridium XI and XVIII, Faecalibacterium, Oscillibacteres, Prevotella, Parabacteroid, unclassified
Nam	2013	16 s-rRNA	The diversity of microbiota was decreased during RT and after RT	Fusobacteria Unclassified	Ruminococcus C.methylpentosum leptom	Firmicutes	Genus: Clostridium sp.BG-C36	–	–	–	–
Manichanh	2008	16 s-rRNA	During and after RT, the diversity in the diarrhoea group was lower than that in patients without diarrhoea and controls	–	–	–	–	Actinobacteria Firmicutes	Class: Bacilli	Firmicutes	Class: Clostridia
Cuzzolin	1992	Bacterial culture	–	Clostridium spp	Clostridium.bistolyticum Clostridium.bifermentans Clostridium.sporogenes	–	Genus: Escherichia coli, Aeromonas hydrophila, Enterococcus faecium 1, Peptococcus Peptostreptococcus spp, Lactobacilli, Fusobacterium Nucleatum total anaerobes	–	–	–	–

*16S rRNA* 16S ribosomal RNA sequencing, *RT* radiation therapy

– Not mentioned in the text

Five studies observed the community composition of the gut microbiota before, during, and after pelvic radiotherapy at different time points and found that some of the microbiota significantly increased while some significantly decreased after radiotherapy (RT) intervention. As shown in Table 3, the microbiota increased or decreased during and after RT compared to those before RT in each study, and the bacterial flora changed at both the phylum level and genus level. The results revealed that the phylum Fusobacteria and other unclassified bacteria were increased after radiotherapy, while Firmicutes and Bacteroidetes were significantly decreased after pelvic radiotherapy. At the order or genus level, *Phascolarctobacterium, Lachnospiraceae, Veillonella, Erysipelotrichaceae, Roseburia, Clostridium*, *Ruminococcus*, *C. methylpentosum*, and *Leptom* were significantly increased, while the relative abundances of other intestinal-dominant genera, such as *Clostridiales, Faecalibacterium*, *Peptococcus* and *Peptostreptococcus*, *Lactobacilli*, *Roseburia*, and other anaerobes, were significantly decreased.

In five studies, the differences in microflora before radiotherapy in patients with RID and those without RID and healthy controls were examined. Prior to radiotherapy, the levels of some Firmicutes, Proteobacteria and Actinobacteria in patients with diarrhoea were higher than those in patients without diarrhoea, while levels of most Firmicutes and Bacteroidetes in patients with diarrhoea were lower than those in patients without diarrhoea. In addition, at the genus or class level, *Sutterella*, *Finegoldia, Peptococcaceae (Clostridia), Prevotella_9, Coprococcus, Desulfovibrio, Bacteroides*, *Veillonella*, *Dialister*, and *Bacilli* were present in patients with diarrhoea, while the levels of intestinally dominant bacteria, such as *Clostridiales, Bacteroides, Blautia, Ruminococcaceae_UCG‐003, Faecalibacterium, Oscillibacteres, Prevotella*, and *Roseburia*, were lower.

## Discussion

Intestinal injury in response to pelvic radiotherapy not only reduces the quality of life of patients but also leads to forced interruption of radiotherapy in severe cases, affecting therapeutic efficiency. Intestinal injury after pelvic radiotherapy includes many symptoms, among which diarrhoea is the most common clinical manifestation and has been a widespread concern [1]. Current studies on the mechanism of diarrhoea following pelvic radiotherapy suggest that it is associated with increased intestinal peristalsis, decreased intestinal immune function [21], intestinal crypt stem cell destruction [22], bile salt malabsorption [23], and disruption of intestinal microbiota homeostasis [19]. In these studies, intestinal epithelial damage due to radiation and disturbance of the intestinal internal environment are generally accepted as important causes of diarrhoea. Relevant studies found that radiation-induced damage to the intestinal epithelium is related to intestinal flora disorder, diarrhoea is unlikely to occur after radiotherapy in germ-free (GF) animals, and the degree of damage to the intestines is mild[24]. Radiotherapy has been linked to the occurrence of intestinal flora disorder. This article reviews currently published studies on the effect of pelvic radiotherapy on intestinal flora, further clarifies which flora are affected by pelvic radiotherapy, and explores the correlation between intestinal flora and radiation-induced diarrhoea.

As the included studies reported, the gut microbiota is disturbed during and after pelvic radiotherapy [13, 16–18]. 16S RNA technology revealed that the diversity of intestinal flora communities at different time points before, during, and after pelvic radiotherapy was significantly decreased. In addition, the diversity of intestinal flora was decreased more significantly in patients who experienced diarrhoea after radiotherapy [13, 16]. These studies provide vital evidence for a link between the alterations in microbiota caused by radiotherapy and postradiotherapy diarrhoea. However, they do not shed light on the mechanistic relationship between microbiota and bowel injury in those who developed postirradiation diarrhoea. GF mice are markedly resistant to lethal radiation enteritis. After a lethal dose of whole-body irradiation in GF mice, apoptosis of endothelial cells and lymphocytes in the villous stromal nucleus of the small intestine was significantly reduced [7]. In addition, when faecal suspensions from diarrhoeic-irradiated mice were transplanted into sterile mice, there was an increase in the release of intestinal inflammatory cytokines (IL-β) in response to irradiation, which was more pronounced than that of GF mice transplanted with normal healthy mouse faeces [9]. These studies revealed that dysbiosis caused by radiation increases the bowel’s susceptibility to injury and may contribute to the development of RID.

In the literature included in this study, five studies reported significant differences in the distribution of bacteria before radiation therapy among patients with radiation-induced diarrhoea, patients without diarrhoea after radiotherapy, and healthy volunteers. On the one hand, the composition of intestinal bacteria before radiotherapy in patients determines whether diarrhoea will occur after radiotherapy, and the microenvironment created by intestinal bacteria and their products is an important factor affecting the occurrence of diarrhoea after radiotherapy. On the other hand, intestinal bacteria change significantly during and after the end of radiotherapy, and changes in these specific species are also responsible for intestinal epithelial damage and diarrhoea. Furthermore, the gut microbiota and its metabolites have a broad and profound influence on multiple aspects of the host gut mucosal immune system [25]. The gut microbiota modulates intestinal immunity through interactions with pattern recognition receptors (PRRs), primarily Toll-like receptors (TLRs). In the intestine, TLRs that are expressed by enterocytes and dendritic cells (DCs) recognize several microbe-associated molecular pattern (MAMP) molecules on the bacterial cell surface, such as lipopolysaccharide (LPS) and peptidoglycan [26]. Dysbiosis due to radiation or other factors can influence both local and systemic immune responses and further induce intestinal damage [27]. Furthermore, pelvic radiotherapy induces bile acid malabsorption (BAM) [23], a vital consequence of diarrhoea. Microorganisms maintain BAM by regulating the metabolism and reabsorption of cholate. Secondary bile acids, formed by gut microbiota from primary bile acids[28], and certain gut microbiota, such as Bacteroides and Lactobacillus, contain cholate hydrolases, which hydrolyse bile salts when intestinal bile salt levels are elevated and play an important role in promoting bile salt metabolism and maintaining intestinal bile salt balance [29, 30]. If the content of these flora is reduced after pelvic radiotherapy so that the bile salts in the intestine cannot be hydrolysed, it can lead to the accumulation of bile salts in the intestine and cause diarrhoea. Although the above studies have explored the mechanism of RID caused by intestinal bacteria, the process of radiation-induced diarrhoea is a complex process, and several questions remain, including what role does intestinal bacteria play in this process? What changes in the intestinal microenvironment are caused by the changes in bacteria? What functions of intestinal epithelial cells are altered by the flora? These all require additional study in the future.

The human gut microbiota can be classified at the phylum level into Bacteroidetes, Actinobacteria, Proteobacteria, Fusobacteria, Firmicutes, and unclassified phyla [31, 32]. Only five of the studies included in our review reported significant phylum-level changes in microbial composition before and after pelvic radiotherapy, although these results were inconsistent. Overall, the consensus observation is a significant increase in Proteobacteria, unclassified bacteria, and Fusobacteria and a significant decrease in Firmicutes and Bacteroidetes in response to pelvic radiotherapy. The phylum diversity in the gut is clearly remodelled after pelvic radiotherapy. This is in contrast to disturbances in the gut microbiota triggered by broad-spectrum antibiotics, which result in a decrease in Firmicutes and an increase in Bacteroidetes [33], suggesting that radiation-induced disturbances of the microbiota have unique characteristics. We also found that the composition of phyla before radiotherapy in patients with diarrhoea was not the same as that in patients without diarrhoea, and Actinobacteria, some Firmicutes and Proteobacteria were significantly increased in patients with diarrhoea. However, in each relevant study, this change was also inconsistent. This discordance was found in the analysis of bacteria, but overall, there was a significant decrease in intestinal predominant beneficial bacteria after pelvic radiotherapy, and patients with diarrhoea displayed less beneficial bacteria in their intestine before radiotherapy than those without diarrhoea. Pelvic radiotherapy clearly kills beneficial intestinal anaerobic bacteria, such as *Faecalibacterium*, Peptostreptococcus, *Lactobacillus*, and *Roseburia*. This significant proportion of the microbiome is less likely to reappear following diarrhoea induced by pelvic radiotherapy. Therefore, probiotic supplementation may reduce the occurrence of diarrhoea in response to pelvic radiotherapy. However, the results of studies on probiotic use for the treatment of radiation-induced diarrhoea are inconsistent. Some studies have demonstrated that probiotic supplements may reduce the occurrence of diarrhoea after pelvic radiotherapy [34–37]. A Cochran systematic review confirmed that probiotic supplementation ameliorated postradiotherapy adverse events [38] but contradicted the results of a previous meta-analysis, which showed that oral probiotic supplementation did not attenuate radiation diarrhoea [39]. Therefore, more studies are needed to further understand the differences in bacterial flora changes after pelvic radiotherapy, the mechanism of bacterial flora disorder leading to diarrhoea, what bacteria should be included in probiotic capsules and how probiotics can positively change the gut microbiome.

## Limitations

There are certain limitations in this systematic review that need to be highlighted. First, all of the included studies were single-centre studies, and the selection of subjects was not reported as random or blind. Furthermore, the selection of a normal healthy control group was not reported, making the subjects not fully representative of the characteristics of the overall population and the selection bias. Second, the included studies were from different countries, and the composition of the gut microbiota may vary by ethnic group [40, 41]. The basic characteristics of the study subjects were not reported in detail, and some studies did not report the number of patients who were lost to follow-up. In addition, the tumour types of pelvic radiotherapy varied among studies, and the existing studies showed that the intestinal flora of patients with different tumour types also varied [42–44]. These factors may therefore contribute to the occurrence of bias. Third, none of the included studies described pelvic radiotherapy techniques or the dose limit of intestinal tissues. With the progress of radiotherapy techniques, different radiotherapy techniques employ different doses for intestinal tissues in pelvic radiotherapy[45–47]. However, the relationship between radiotherapy dose and changes in bacterial flora could not be clarified from these studies. In addition, due to the small number of included studies and inconsistently reported changes in the composition of the intestinal microbiota, it was not possible to perform a meta-quantitative analysis of changes in the microbiota.

## Conclusions

The studies we reviewed demonstrated that pelvic radiotherapy can lead to a decrease in intestinal flora diversity with a consequent change in community composition, and this decrease is more obvious in patients who develop diarrhoea in response to radiotherapy. In addition, the composition of the gut microbiota before radiotherapy was different between patients with postradiotherapy diarrhoea and those without diarrhoea. However, relevant studies have not reported consistent results regarding the changes in microbiota composition. Overall, our study reveals the correlation between intestinal flora and radiation-induced diarrhoea, providing a certain basis for the causes of diarrhoea in patients undergoing pelvic radiotherapy, but the causal relationship and mechanism between intestinal flora and disease has not been determined, which should be the focus of future research efforts.

## Data Availability

The datasets used and/or analysed during the current study are available from the corresponding author on reasonable request.

## Appendix

The literature search strategy with the example of Pubmed

## Abbreviations

RE: Radiation enteropathy
PRISMA: Systematic reviews and meta-analysis
PROSPERO: Prospective register of systematic reviews
RCTs: Randomized controlled trials
RT: Radiotherapy
GF: Germ-free
PRRs: Pattern recognition receptors
TLRs: Toll-like receptors
DCs: Dendritic cells
MAMP: Microbe-associated molecular pattern
LPS: Lipopolysaccharide
BAM: Bile acid malabsorption
5FU: 5-Fluorouracil
